# The impact of childhood psychological maltreatment on mental health outcomes in adulthood: a protocol for a systematic review and meta-analysis

**DOI:** 10.1186/s13643-021-01777-4

**Published:** 2021-08-12

**Authors:** Zhuoni Xiao, Mina Murat Baldwin, Franziska Meinck, Ingrid Obsuth, Aja Louise Murray

**Affiliations:** 1 Department of Psychology, University of Edinburgh, 7 George Square, Edinburgh, EH8 9JZ UK; 2 School of Social and Political Science, University of Edinburgh, Edinburgh, UK; 3 Faculty of Health Sciences, North-West University, Vanderbijlpark, South Africa; 4 Clinical Psychology Department, University of Edinburgh, Edinburgh, UK

## Abstract

**Background:**

Research suggests that childhood psychological maltreatment (i.e., emotional abuse and emotional neglect) is associated with mental health problems that persist into adulthood, for example anxiety, depression, post-traumatic stress disorder (PTSD), suicidal ideation, and aggression; however, a systematic review and meta-analysis of the existing literature would help clarify the magnitude and moderators of these associations, and the extent to which they may be affected by publication bias, as well as the methodological strengths and weakness of studies in this area.

**Method:**

The reporting of this protocol follows the Preferred Reporting Items for Systematic Review and Meta-Analysis Protocols (PRISMA-P) Statement. Searches will be carried out via several databases, including Web of Science, Medline, PubMed, PsycINFO, Applied Social Science Index and Abstract, ERIC and EMBASE. Empirical peer-reviewed research articles that fit pre-specified eligibility criteria will be included in the review. Studies will be eligible if they include participants age 18 or over at time of mental health assessment, include information on childhood psychological maltreatment (emotional abuse and/or neglect) perpetrated by a primary caregiver or adult in the same household, and provide quantitative information on the association between these factors. Studies using prospective and retrospective designs and written in either English or Chinese will be eligible. Two independent reviewers will screen and assess studies for inclusion in the review as well as extract the data, with consensus reached through discussion in cases of discrepancy. A third reviewer will be consulted to resolve any discrepancies that remain. The relevant Newcastle–Ottawa scales will be used for assessing the quality of studies. If a sufficient number of comparable studies are retrieved, a meta-analysis will be conducted using a random effects model. Study-level moderators (i.e., year of publication, quality of the study and study geographical location) will be examined in the meta-analyses.

**Discussion:**

This systematic review will provide an understanding of the long-term effects of childhood psychological maltreatment on adult mental health, which adds to previous reviews focusing primarily on the effects of physical and sexual abuse. The results of the review will help inform clinical practice in approaches to treating those with a history of psychological maltreatment in childhood. The gaps and weaknesses in the evidence identified will also inform recommendations for future research.

## Background

Childhood abuse is significantly associated with adverse emotional, cognitive, behavioural and social outcomes for children [16, 19, 22], with difficulties frequently continuing into adulthood [7]. According to the World Health Organization (WHO) (2020), childhood abuse refers to all forms of abuse (e.g. physical, sexual, emotional, psychological and neglect) that result in potential or actual harm to a child’s physical or psychological health.

Childhood emotional abuse is the type of abuse least well-studied [1]. There has been one systematic review on the association between childhood emotional abuse and neglect in school-aged children [19],however, there has been no systematic review or meta-analysis on the long-term mental health effects of childhood psychological maltreatment on adults.

There are different definitions of psychological maltreatment, for example, Vega Castelo (2012) stated that psychological maltreatment refers to affective and cognitive aspects of child maltreatment. For the purpose of this review, psychological maltreatment is defined as including two specific concepts: childhood emotional abuse and childhood emotional neglect. Forms of psychological maltreatment may include rejecting, isolating, neglecting, exploiting, and terrorizing [12]. Emotional abuse in childhood refers to continual deliberate mistreatment of a child, which may include deliberately trying to scare, humiliate, ignore, and isolate the child. Emotional abuse is often a part of other forms of abuse,however, it can also happen on its own [4]. In contrast to emotional abuse, emotional neglect may be unintentional, and caregivers are sometimes unaware that they are emotionally neglecting their child. Emotional neglect in childhood refers to caregivers’ failure to recognize, understand or provide what a child really needs, and may sometimes refer to lack of attention to a child [4]. The primary distinction between childhood emotional neglect and childhood emotional abuse is that the former reflects indifferent parenting while the latter reflects hostile parenting [17].

This review will focus on psychological maltreatment perpetrated by primary caregivers or another adult in household specifically. This focus is motivated by the fact that in the traditional family model, primary caregivers and cohabiting adults are often the most important figures for a child. This is also reflected in commonly used measures of maltreatment. For example, in measures such as the Childhood Traumatic Questionnaire [5], Adverse Childhood Experience, etc., the items ask whether primary caregivers or adults living in the same household committed maltreatment. The focus on psychological maltreatment is motivated by the fact that it is currently the least-well studied form of abuse in terms of its effects on adult mental health. Part of the reason may be the challenges inherent in measuring psychological maltreatment. Compared with physical and sexual abuse, the assessment and identification of psychological maltreatment can be more difficult [2], since there is no physical evidence of its occurrence. However, the negative outcomes of it may manifest in numerous ways such as impaired emotional, cognitive, or social development, including outcomes such as depression [13], helplessness (Black, SlepAM, & Heyman, 2001), aggression (Diza, Simantov, & Rickert, 2002), emotional dysregulation (Burns, Jacksons, & Harding, 2010) delinquency, substance abuse, PTSD, anxiety, and low self-esteem (Kilpatrict, Saunders, & Smith, 2003).

### Rationale for the current review

There are numerous systematic reviews on the associations between physical or sexual abuse and adult mental health [3, 15],however—to the best of the authors’ knowledge—to date, no research has been carried out to synthesize current evidence on the relationships between childhood psychological maltreatment by primary caregivers (or adults living in the same household) and adult mental health. A systematic review on this topic can provide an understanding of the consistency and strength of the link between early childhood maltreatment and adult mental health outcomes at both the clinical and sub-clinical level. A systematic review and meta-analysis can help provide a more precise estimate of the association than has been provided by primary studies to date. It will also allow us to examine the factors that moderate the magnitude of this association, and to evaluate whether the field is affected by publication bias. Further, it will provide a characterization of the quality of empirical studies in this field and identify gaps in the literature.

The primary review questions will be:

- What are the long-term effects of childhood psychological maltreatment on adult mental health?
- What are the unique effects of childhood psychological maltreatment by caregivers on adult mental health after adjusting for other forms of abuse?
- How do study-level moderators such as year of publication, quality of study and location of study affect these associations?

## Method

The Preferred Reporting Items for Systematic Review and Meta-Analysis Protocols (PRISMA-P) recommendations have been used to guide the reporting in this systematic review protocol and will be used to guide the reporting of the review itself [21]. This systematic review protocol has been registered in the International Prospective Register of Systematic Reviews (PROSPERO) with registration number CRD42020197833.

### Search strategy

To search the existing literature on childhood psychological maltreatment, the following keywords will be used: ‘child abuse’, ‘childhood psychological maltreatment’, ‘childhood emotional abuse’, ‘child neglect’, ‘childhood emotional neglect’, ‘psychological aggression’, ‘psychological violence’, ‘psychological domestic violence’ and ‘childhood psychological victimisation’. The Boolean operator ‘OR’ will be used to combine the search terms and with specific syntax be adapted to the different databases.

To capture the concept of mental health, these key search terms will be used: ‘mental health’, ‘generalised anxiety disorder’, ‘depression’, ‘major depression disorder’, ‘PTSD’, ‘personality disorder’, ‘eating disorder’, ‘bipolar disorder’, ‘schizophrenia’, ‘panic disorder’, ‘psychosis’, ‘social anxiety disorder’, ‘suicide ideation’, ‘suicide attempt’, ‘non-suicidal self-injury’ and ‘substance abuse’. The Boolean operator ‘OR’ will be used to combine these search terms, adapted to the syntax of different databases.

The Boolean operator (‘AND’) will be used to combine keywords from psychological maltreatment and mental health. In addition, the maltreatment terms will be combined with child* and the mental health terms with adult* using the AND operator in order to link the concepts to the relevant developmental stages.

Several databases will be used searching for relevant papers: Web of Science, Medline, PubMed, PsycINFO, Applied Social Science Index and Abstract, ERIC and EMBASE. For grey literature, several databases will be used: WHO database, PhD thesis/dissertation databases, and Open Grey.

For literature written in Chinese, ZhiWang which is a well-known database in China, and covers various journals written in Chinese, will be used for searching the literature.

### Inclusion criteria

- Participants aged over 18 at assessment of the mental health problems.

- Measurement of abuse: studies that measured childhood psychological maltreatment using retrospective self-report, questionnaires, interviews, or police or social work records will be included.
- Measurement of mental health: studies that measured mental health problems (standard diagnoses as listed in the DSM-V or ICD-10 or using mental health scores based on validated measures) using self-report, questionnaires or clinical interviews will be included.
- Types of maltreatment: studies that only assessed childhood psychological maltreatment, childhood emotional neglect, childhood emotional abuse, or studies that assessed both childhood emotional abuse and childhood emotional neglect and other types of abuse (e.g., physical, or sexual) will be included. The abuse must have been committed by the primary caregivers, or the adult living in the same household.
- Comparison: adults who experienced only childhood psychological maltreatment versus adults who experienced different forms of abuse during childhood, with or without psychological maltreatment versus adults who experienced no abuse during childhood will be compared.
- Ascertainment of exposure to childhood psychological maltreatment by primary caregivers (or adults living in the same household): Studies using either retrospective or prospective data will be included.
- Studies that reported odds ratio or other effect size: If the studies do not report the relevant effect size, they will be eligible for inclusion only if they provide the raw information such that the effect size could be calculated. When the raw information not available in the text, authors will contact the authors to request such data.
- Additional inclusion criteria include:
- Articles written in either English or Chinese will be included reflecting the language capabilities of the team.

### Exclusion criteria

- Any book chapters, case studies, letter, opinions, and editorials that do not present new data will be excluded.
- Qualitative investigations will be excluded.
- Studies that do not provide an analysis of childhood psychological maltreatment will be excluded.
- Studies that focus on psychological maltreatment perpetrated by non-parental others or where data for primary caregivers or another adult in household cannot be disaggregated from data on abuse perpetrated by others will not be included.
- Studies where different types of abuse are combined and not separately reported so that it is not possible to obtain an effect for childhood psychological maltreatment will be excluded.
- Studies where the outcome is physical rather than mental illness will be excluded.
- Review papers (narrative reviews, systematic reviews, and meta-analyses) will be excluded.

### Study selection

**Fig. 1:**
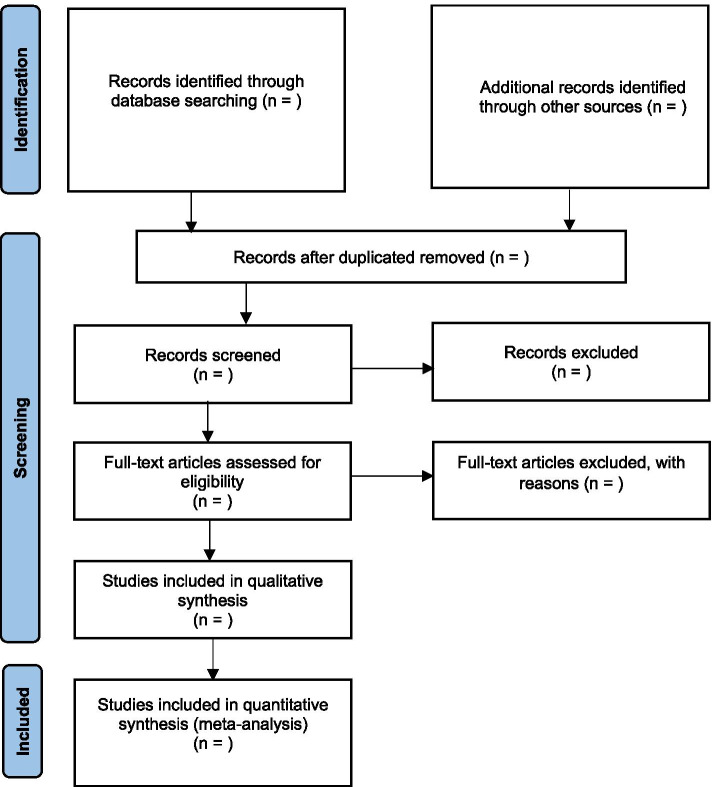
PRISMA flow diagram

The bibliographic software program Zotero will be used to manage and store relevant studies. Duplicate studies will be removed via this software. Two independent reviewers will scrutinise the electronic searches for eligibility and inclusion of studies into the systematic review based on their title and abstract. Full texts of potentially relevant papers will be retrieved and reviewed independently by two reviewers. A final determination of whether an article meets inclusion criteria will be made on examination of the full article, the reason for each excluded study will be documented. A third reviewer will be consulted to resolve any discrepancies that cannot be resolved through discussion between the original reviewers. Figure 1 presents the flow diagram to be adopted in the systematic review for study selection [20].

### Methodological appraisal of study

Classification of risk of bias as recommended by the Newcastle–Ottawa Quality Assessment Scale will be used to assess the quality of selected case–control for retrospective study or cohort studies for longitudinal studies [25]. Main domains of this assessment are selection (adequateness of case definition, representativeness of the cases, selection of controls and definition of controls), comparability (comparability of cases and controls based on the design or analysis) and exposure (ascertainment of exposure, same method of ascertainment for cases and control and non-response rate). A study can be awarded a maximum of four stars for selection, two stars for comparability and three stars for exposure. More stars represent lower risk of bias. Two reviewers will independently assess the studies for methodological quality with discrepancies being resolved through discussion and a third reviewer will be consulted where consensus cannot be reached through discussion.

### Data extraction

Study findings will be extracted using a structured database. It will include pertinent information such as author name and date of publication, sample size, sample population, study geographical location, sample population demographic, study setting, study methodology, types of abuse, measurement of childhood psychological maltreatment, duration of abuse, measurement of various mental health outcomes, perpetrator of the maltreatment, age at exposure to maltreatment, the relation between childhood psychological maltreatment and mental health outcomes (as an odds ratio or risk ratio), and covariates adjusted for. When available, both adjusted and unadjusted statistics will be extracted. Two reviewers will independently conduct the data extraction with consensus reached through discussion in case of discrepancies. Where consensus is not reached through discussion, a third reviewer will be consulted. If any new categories are identified during the course of the review, they will be added, and the extraction database will be modified as needed. If there are any missing data or relevant information, authors will be contacted to supply the information. To detect the unique effects of childhood psychological maltreatment by caregivers on adult mental health after adjusting for other forms of abuse, researchers will extract the statistical information of the studies exploring the associations between childhood psychological maltreatment and adult mental health when adjusting for other types of abuse.

### Data analysis

A narrative synthesis of the findings from the included studies will be presented. The narrative synthesis will focus on socio-demographic characteristics of the samples (duration of abuse, who the maltreatment was inflicted by, age at exposure to maltreatment), characteristic of the studies (study setting, sample size, study design), methodology (questionnaire, self-report, experimental design, clinical interview, police or social work records), types of mental health issues, effect size and odd/risk ratios.

A meta-analysis will be conducted if there are enough studies with information related to both childhood psychological maltreatment and mental health. Results will be summarized using a forest plot. Results from different study designs will not be pooled together (e.g., studies that assessed only childhood psychological maltreatment and studies that assessed different types of abuse) to prevent a misleading summary of the study effect; rather, they will be analysed separately. If possible, meta-analyses of both adjusted and unadjusted effects will be conducted and results compared. A random effects model will be utilized for the meta-analysis as it is likely that studies will not be homogeneous. Studies are expected to represent fairly substantial differences in method (i.e. types of participants, measurements) and are thus not anticipated to reflect a single underlying effect size. The ‘Metafor’ package for R statistical software will be used for meta-analysis [24].

The GRADE criteria will be used to assess the quality of the evidence provided by the observational studies in relation to the outcome (Higgins & Green, 2011). The quality of the evidence will be rated as very low, low, moderate, and high; and factors that may decrease the quality are risk of bias, imprecision, inconsistency and indirectness (Higgins & Green, 2011).

### Assessment of heterogeneity and moderator analysis

Study heterogeneity will be assessed by examining the characteristics of studies and similarities between childhood psychological maltreatment and mental health outcomes. Statistical heterogeneity will be assessed by calculating *Q* and *I*^2^. Where there are sufficient numbers of studies in the meta-analysis, study-level moderators will be tested. These may include study quality (based on the quality assessment described above), study geographical location, year of publication, and sample size. Moderator analysis will be using the ‘Metafor’ package.

### Assessment of reporting bias

In case of an appropriate number of studies (*n* ≥ 10), publication bias will be assessed using a funnel plot for each outcome by plotting the effect size against study size (Higgins & Green, 2011). An Egger test [11] and the trim and fill method [10] will be used to statistically test for publication bias and its potential impact.

## Discussion

This protocol outlines the plan for a systematic review and, if applicable, a meta-analysis on the effects of childhood emotional abuse and childhood emotional neglect (collectively ‘psychological maltreatment’) perpetrated by primary caregivers or adults living in the same household in childhood on adult mental health outcomes. There is currently no systematic review and meta-analysis focusing specifically on the long-term effects of childhood psychological maltreatment on adult mental health outcomes, therefore, the review will help fill this important gap. The findings from this review could help illuminate the long-term impact of psychological maltreatment, in combination with and net of other forms of abuse. This can help inform prevention and intervention strategies to help target resources and minimise the impact of psychological maltreatment. It will also potentially provide insights into whether the impact of psychological maltreatment varies across contexts; which mental health outcomes it is most strongly related to; and whether its impact has changed over time. This review will also explore where the major gaps are in current evidence in other to make recommendations for future research. Finally, it will help provide an assessment of the quality of the work on the field and identify areas for improvement in future research to strengthen the evidence in the field.

## Data Availability

Not applicable.

## Abbreviations

DSM-V: The Diagnostic and Statistical Manual of Mental Disorders (5th edition)
ICD-10: International Classification of Diseases, tenth revision
PRISMA: Preferred Reporting Items for Systematic Review and Meta-Analysis
PTSD: Post-traumatic stress disorder
WHO: World Health Organization
